# Effects of co-occurring *Wolbachia* and *Spiroplasma* endosymbionts on the *Drosophila* immune response against insect pathogenic and non-pathogenic bacteria

**DOI:** 10.1186/s12866-016-0634-6

**Published:** 2016-02-09

**Authors:** Upasana Shokal, Shruti Yadav, Jaishri Atri, Julia Accetta, Eric Kenney, Katherine Banks, Akash Katakam, John Jaenike, Ioannis Eleftherianos

**Affiliations:** Insect Infection and Immunity Lab, Department of Biological Sciences, Institute for Biomedical Sciences, The George Washington University, 5675 Science and Engineering Hall, 800 22nd Street NW, Washington, D.C. 20052 USA; Department of Biology, University of Rochester, Rochester, NY 14627 USA

**Keywords:** *Drosophila melanogaster*, Endosymbionts, *Wolbachia*, *Spiroplasma*, Insect pathogen, *Photorhabdus luminescens*, Infection, Innate immunity, Host-microbe interactions

## Abstract

**Background:**

Symbiotic interactions between microbes and animals are common in nature. Symbiotic organisms are particularly common in insects and, in some cases, they may protect their hosts from pathogenic infections. *Wolbachia* and *Spiroplasma* endosymbionts naturally inhabit various insects including *Drosophila melanogaster* fruit flies. Therefore, this symbiotic association is considered an excellent model to investigate whether endosymbiotic bacteria participate in host immune processes against certain pathogens. Here we have investigated whether the presence of *Wolbachia* alone or together with *Spiroplasma* endosymbionts in *D. melanogaster* adult flies affects the immune response against the virulent insect pathogen *Photorhabdus luminescens* and against non-pathogenic *Escherichia coli* bacteria.

**Results:**

We found that *D. melanogaster* flies carrying no endosymbionts, those carrying both *Wolbachia* and *Spiroplasma*, and those containing *Wolbachia* only had similar survival rates after infection with *P. luminescens* or *Escherichia coli* bacteria. However, flies carrying both endosymbionts or *Wolbachia* only contained higher numbers of *E. coli* cells at early time-points post infection than flies without endosymbiotic bacteria. Interestingly, flies containing *Wolbachia* only had lower titers of this endosymbiont upon infection with the pathogen *P. luminescens* than uninfected flies of the same strain. We further found that the presence of *Wolbachia* and *Spiroplasma* in *D. melanogaster* up-regulated certain immune-related genes upon infection with *P. luminescens* or *E. coli* bacteria, but it failed to alter the phagocytic ability of the flies toward *E. coli* inactive bioparticles.

**Conclusion:**

Our results suggest that the presence of *Wolbachia* and *Spiroplasma* in *D. melanogaster* can modulate immune signaling against infection by certain insect pathogenic and non-pathogenic bacteria. Results from such studies are important for understanding the molecular basis of the interactions between endosymbiotic bacteria of insects and exogenous microbes.

## Background

The insect innate immune system includes a variety of defense mechanisms that individually or in tandem prevent foreign microoorganisms from invading the insect body or suppressing pathogen growth and proliferation [1, 2]. The main insect defense mechanisms include the expression of antimicrobial peptides (AMP) by the fat body (equivalent to mammalian liver) into the hemolymph (equivalent to mammalian blood) [3], cellular responses by insect hemocytes (equivalent to mammalian white blood cells) [4], melanization and coagulation responses in the hemolymph [5], and generation of reactive oxygen species and nitric oxide in epithelial cells [6, 7]. These immune functions have been well characterized in the common fruit fly *Drosophila melanogaster*, which is an excellent model for studying the molecular and functional basis of the innate immune response [8, 9].

In addition to interactions with exogenous microbes, insects also interact with their endosymbiotic bacteria that are able to manipulate various physiological functions and the reproductive properties of their hosts [10]. Two of the most widespread and widely studied endosymbionts are *Wolbachia* and *Spiroplasma*, the first are carried by between 20 % and 70 % of all insect species [11, 12]. *Wolbachia* is a genus of maternally-transmitted, intracellular, Gram-negative, α-proteobacteria which is known to induce parthenogenesis, male-killing, feminization, and cytoplasmic incompatibility (sperm-egg incompatibility) in their insect hosts [13, 14]. *Spiroplasma* is a genus of wall-less, motile, helical, Gram-positive bacteria which interact endo- and extracellularly with various insect species. Some strains of *Spiroplasma* can cause female-biased sex ratios through selective death of male offspring in their insect hosts [15]. *Wolbachia* and *Spiroplasma* have developed strategies to evade inherent insect host immune defenses in order to ensure survival and transmission and as a result, insect hosts have accordingly developed mechanisms to regulate endosymbiont populations to prevent fitness costs [16–19].

Although relationships between insects and their endosymbiotic bacteria have been studied most commonly with respect to nutritional effects on the host, recent studies have shown that some endosymbionts can protect their insect hosts from infections by certain pathogens [20]. Interestingly, *D. melanogaster* flies naturally contain *Wolbachia* and *Spiroplasma* endosymbionts, and they therefore constitute a convenient experimental model to investigate the impact of endosymbionts on the regulation of host immune function [12, 21]. Previous studies have shown that the presence of certain *Wolbachia* strains in *D. melanogaster* flies although it does not alter immune gene transcription [22], it can greatly enhance survival to infection by certain RNA viruses [23–31] and parasitoid wasps [32–34], but not to bacterial infections [35, 36]. The presence of *Spiroplasma* endosymbionts in *D. melanogaster* flies does not activate the immune system, but induction of Toll or immune deficiency (Imd) immune signaling increases *Spiroplasma* titer in the fly hemolymph. Also, *D. melanogaster* flies carrying *Spiroplasma* endosymbionts are more sensitive to some Gram-negative bacterial pathogens [37].

The *Photorhabdus* genus of entomopathogenic bacteria is a member of the *Enterobacteriaceae* family. In addition to being a highly virulent pathogen of insects, *Photorhabdus* maintains a mutualistic relationship with nematodes in the *Heterorhabditidiae* family [38]*. P. luminescens* bacteria are found in the gut of the infective juvenile (IJ) stage of the nematode *Heterorhabditis bacteriophora* [39]. The IJ stage is an obligate part of the nematode life-cycle that is required for infection of various insect species. Once inside the insect, the IJ regurgitates *P. luminescens* into the hemolymph where the bacteria begin to divide exponentially producing a wide range of toxins and hydrolytic enzymes that result in insect death within a few days [40]. Previous studies have shown that *P. luminescens* has evolved strategies for actively suppressing humoral and cellular immunity in infected insects to facilitate its successful replication and spread into the host [41].

The goal of this study was to investigate for the first time the participation of *Wolbachia* endosymbionts alone or in combination with *Spiroplasma* in the immune response of *D. melanogaster* adult flies against the virulent insect pathogen *P. luminescens* and a non-pathogenic strain of *Escherichia coli*. For this, we used *D. melanogaster* strains carrying different combinations of *Wolbachia* and *Spiroplasma* endosymbionts and found that although the survival response of flies with or without endosymbionts was unaffected upon infection with the pathogen *P. luminescens* or the non-pathogen *E. coli*, there were changes in bacterial load and endosymbiont titers in the infected flies. Interestingly, we further found significant induction of certain immune-related genes in flies carrying both endosymbionts following infection with the pathogenic or the non-pathogenic bacteria. This study shows that *D. melanogaster* and its endosymbiotic microbes form a particularly useful system to understand the impact of endosymbiosis on host immune activation and function against virulent pathogens as well as against non-pathogenic microorganisms.

## Methods

### Fly strains

*D. melanogaster* flies carrying both *Wolbachia pipientis* (strain *w*Mel) and *Spiroplasma poulsonii* (strain MSRO) endosymbionts (designated as W + S+), no endosymbiotic bacteria (W-S-), or *Wolbachia* only (W + S-) were used in all experiments. All three types were derived from a single isofemale line collected in Uganda by John Pool. The original wild-caught female was infected with both endosymbionts (W + S+); a sub-strain carrying only *Wolbachia* (W + S-) was obtained as a result of imperfect maternal transmission of *Spiroplasma* during lab culture; and a symbiont-free sub-strain (W-S-) was obtained by raising flies on medium containing tetracycline (final concentration of 0.25 mg/mL media) for one generations, followed by >10 generations of growth on tetracycline-free medium to allow recovery from tetracycline treatment. Because *Spiroplasma* is a male-killer [42], W + S+ strain produces a few or no males; so maintaining this strain entails mating its females to males from the W-S- strain every generation. All *D. melanogaster* strains were amplified for experimentation with approximately 2.5 g of Carolina Formula 4-24 Instant *Drosophila* media (Carolina Biological Supply), 10 mL of deionized water, and a dash (approximately 0.003 g) of dry baker’s yeast granules. All stocks were maintained at 25 °C and a 12:12-h light:dark photoperiodic cycle. Adult female flies aged 7-10 day old were used in infection assays with bacteria.

### Endosymbiont status of fly strains

Presence of endosymbionts in *D. melanogaster* strains was confirmed by performing diagnostic PCR on at least ten individual flies per strain. DNA from flies was isolated using DNeasy Blood & Tissue Kit (Qiagen). PCR amplifications of *Wolbachia* and *Spiroplasma* sequences were performed using the following sets of primers: Wsp (*Wolbachia*), Forward: CATTGGTGTTGGTGTTGGTG and Reverse: ACCGAAATAACGAGCTCCAG [43]; and DnaA (*Spiroplasma*), Forward: TTAAGAGCAGTTTCAAAATCGGG and Reverse: TGAAAAAAACAAACAACAAATTGTTATTACTTC [44] Each reaction was carried out in 50 μl volume containing 20 μl of 5-Prime Hot Master mix, 1 μl each of forward and reverse primer (10 μM), 27 μl of nuclease free water and 1 μl of DNA (100–300 ng/μl). PCR amplifications were performed using a Bio-Rad T100 Thermal Cycler with the following cycling conditions: 94 °C for 2 min, 34 cycles of 94 °C for 30 s, 59 °C (Wsp primers) or 56.7 °C (DnaA primers) for 1 min and 72 °C for 30 s, and 72 °C for 5 min. PCR control reactions for ribosopmal protein L32 (*RpL32*) gene were performed using the primers Rp49, Forward: GATGACCATCCGCCCAGCA and Reverse: CGGACCGACAGCTGCTTGGC with annealing temperature of 61 °C [45]. PCR samples were run on a 0.8 % agarose gel DNA bands were visualized using a Molecular Image Chemidoc XRS (Bio-Rad).

### Bacterial strains

The insect pathogenic bacterium *P. luminescens* subsp. laumondii (strain TT01) and the non-pathogenic bacterium *Escherichia coli* (strain K12) were used for fly infections. Bacterial cultures (10 ml) were prepared in sterile Luria-Bertani (LB) broth and grown for approximately 18–24 h at 30 °C on a rotary shaker at 280 rpm. Bacterial cultures were centrifuged at 885 g or 3,000 rpm and 4 °C for 5 min and the resulting bacterial pellets were washed and re-suspended in 1X sterile phosphate-buffered saline (PBS, Sigma Aldrich). Bacterial cell concentration of the final solution was analyzed using a spectrophotometer (NanoDropTM 2000c – Thermo Fisher Scientific), and concentration was adjusted to Optical Density (260 nm) of 0.1, for *P. luminescens* and 0.015 for *E. coli.*

### Fly survival

Fly infections were carried out by injection of 18.4 nl of a bacterial suspension (*P. luminescens* or *E. coli*) using a Nanoject II apparatus (Drummond Scientific) equipped with glass capillaries prepared with the use of a Micropipette Puller (Sutter Instruments). Flies were handled using a stereomicroscope outfitted with lights and the Ultimate Flypad (Flystuff). Injections were performed into the thorax of 7–10 day old adult flies that were previously anesthetized briefly with carbon dioxide. Injection of the same volume of PBS was used as a control. After infections, flies from each *D. melanogaster* strain were transferred to fresh vials with instant media at 25 °C and survival was scored at 6-h intervals and up to 1 day. Two replicates of ten flies were used for each treatment and each assay was replicated three times.

### Cloning of plasmid DNA and generation of standard curves

DNA from *Wolbachia* and *Spiroplasma* was extracted from 7–10 day old uninfected W + S+ flies. DNA from *E. coli* and *P. luminescens* was extracted from bacterial overnight cultures. DNA samples were isolated using the Dneasy Blood and Tissue kit (Qiagen) and PCR amplifications were performed using the primers Mcf-1 (*P. luminescens*), Forward: TTGGCGGGGTGGTAGTCG and Reverse: CAGTTCAGCTTCCTTCTCTAA; 16S rRNA (*E. coli*), Forward: GGAAGAAGCTTGCTTCTTTGCTGAC and Reverse: AGCCCGGGGATTTCACATCTGACTTA; as well as Wsp and DnaA primers with the mix conditions that were described above. PCR amplifications for *Mcf-1* and *16 s rRNA* sequences were performed using the following cycling conditions: 94 °C for 2 min, 34 cycles of 94 °C for 30 s, 61 °C for 1 min and 72 °C for 30 s, and 72 °C for 5 min. The PCR products were cloned into Strataclone cloning vector (Agilent Technologies) and *E. coli* competent cells were transformed and then grown overnight in LB broth at 37 °C. Plasmids were isolated using GenElute™ Plasmid Miniprep Kit (Sigma Aldrich) and then eluted in 40 μl of nuclease-free water. Dilutions were made for each plasmid to generate the standard curves. The reactions contained 5 μl of the diluted plasmid with 10 μl of SYBR® GreenER with Premixed ROX (Invitrogen), 4.2 μl of nuclease free water and 10 pmol of each forward and reverse primer. The generated standard curves were used for estimating bacterial load (Colony Forming Units, CFU) and endosymbiont titers.

### Bacterial load and endosymbiont titers

Five adult flies from each strain were injected with *E. coli*, *P. luminescens*, or 1x sterile PBS (septic-injury control), and then frozen at 0, 6 and 18 h post infection. DNA samples were eluted in 40 μl of elution buffer and concentrations were measured using a NanoDrop. Each PCR reaction included 10 μl of EXPRESS SYBR® GreenER with Premixed ROX (Invitrogen), 10 μM of each forward and reverse primer sets (Mcf-1, 16 s rRNA, Wsp and DnaA) and 350 ng of each DNA sample. Cycling conditions for estimating *E. coli* and *P. luminescens* load were 50 °C for 2 min, 95 °C for 2 min, 40 cycles of 95 °C for 15 s and an annealing step of 61 °C for 15 s. Cycling conditions for estimating *Spiroplasma* titers were the same except for the annealing step which was 56.7 °C. For estimating *Wolbachia* titers, cycling conditions were 50 °C for 2 min, 95 °C for 10 min, 40 cycles of 95 °C for 30 s, 59 °C for 1 min, and 72 °C for 30 s. All samples were run in duplicates and the experiments were repeated three times.

### Gene trascription

Four adult flies from each strain were injected with *P. luminescens* TT01, *E. coli* K12 or PBS, and frozen at 0, 6, and 18 h after infection. Total RNA was extracted using the PrepEase RNA spin kit (Affymetrix USB) and samples were suspended in 40 μL of sterile nuclease-free water. Complementary DNA (cDNA) synthesis was carried out using the High Capacity cDNA Reverse Transcription Kit (Applied Biosystems, USA), 1 μl of Recombinant RNasin® Ribonuclease Inhibitor (Promega, USA) and 140 ng of RNA sample as starting material in a total reaction volume of 20 μl. PCR cycles included 25 °C for 10 min, 37 °C for 2 h, and 85 °C for 5 min. Resulting cDNA samples were diluted 1:10 in nuclease-free water and 1 μl was used as a template for quantitative RT-PCR experiments using the EXPRESS SYBR® GreenER kit with Premixed ROX (Invitrogen) on a Mastercycler® ep realplex^2^ (Eppendorf). The reactions were carried out in a total reaction volume of 20 μl and technical duplicates were run for each sample and set of primers (Table 1). The cycling program was: 50 °C for 2 min, 95 °C for 2 min, 40 cycles of 95 °C for 15 s and an annealing step for 15 s. For each sample, the amount of mRNA detected was normalized to mRNA values of the control housekeeping gene *RpL32*. Normalized data were used to quantify the relative level of a given mRNA according to cycling threshold analysis (ΔCt), and the data were expressed as the ratio of 2^CT(RpL32)^/ 2^CT(gene)^. Data are presented as the ratio of infected flies to PBS injected flies (negative controls for bacterial infections). Results represent the mean values and standard deviations of relative values from three biological repetitions.

**Table 1:** Primers used for qRT-PCR analysis

Gene	Accession No	Primer	Sequence	Tm (°C)
*Cecropin-A1*	CG1365	Forward	TCTTCGTTTTCGTCGCTCTC	60
		Reverse	CTTGTTGAGCGATTCCCAGT	
*Defensin*	CG1385	Forward	CGCATAGAAGCGAGCCACATG	56
		Reverse	GCAGTAGCCGCCTTTGAACC	
*Turandot M*	CG14027	Forward	GCTGGGAAAGGTAAATGCTG	61
		Reverse	AGGCGCTGTTTTTCTGTGAC	
*Puckered*	CG7850	Forward	GGCCTACAAGCTGGTGAAAG	61
		Reverse	AGTTCAGATTGGGCGAGATG	
*RpL32*	CG7939	Forward	GATGACCATCCGCCCAGCA	61
		Reverse	CGGACCGACAGCTGCTTGGC	

All sequences read 5′ to 3′ left to right

### Hemocyte phagocytosis

Adult flies were injected in the abdomen with 15.6 nl of 1 mg/ml lipophilized pHrodo-labeled *E. coli* particles (Molecular Probes, P35361) reconstituted in 1 ml sterile, Diethylpyrocarbonate (DEPC) treated water. Injected flies were then affixed dorsally/wing side down to a glass microscope slide using clear nail polish and living flies were observed at 30, 45, and 60 min post infection using a fluorescent microscope outfitted with a pHrodo red filter at 10x magnification. Resulting images were processed using ImageJ software and background fluorescence was measured. Relative amounts of fluorescence were measured by applying Shanbhag thresholding to images and measuring the resulting area, mean fluorescence of background and integrated density. Corrected total fluorescence was determined using the following equation: Corrected total fluorescence = Integrated Density – (Area * Mean fluorescence of background). Data were statistically analyzed via GraphPad Prism5 software and the Chi square test. Each experiment was repeated three times with 6-12 flies per treatment.

### Statistical analysis

Statistics were performed using the GraphPad Prism5 software. Statistical analysis of data from survival experiments was conducted using a log-rank (Mantel-Cox) and Chi square tests. *P* values below 0.05 were considered statistically significant. Unpaired two-tailed *t*-test was performed for analyzing bacterial load and endosymbiont titers. Means were compared using a one-way analysis of variance (ANOVA) with a Tukey post-hoc test for multiple comparisons. All figures were also generated using GraphPad Prism5 software.

## Results

### Presence of *Wolbachia* and *Spiroplasma* in *D. melanogaster* strains

**Fig. 1:**
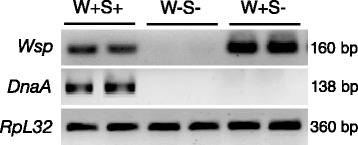
PCR diagnostic for the presence or absence of endosymbionts in *D. melanogaster* strains. Amplification of *Wsp* and *DnaA* gene sequences from 7-10 day old flies carrying both *Wolbachia* and *Spiroplasma* (W + S+), flies lacking both endosymbionts (W-S-), and flies carrying *Wolbachia* only (W + S-). The sizes of the PCR products are indicated. Two replicate experiments (different flies) are shown for each *D. melanogaster* strain. PCR amplification of the constitutively expressed gene *RpL32* is used as loading control

We used PCR to document the presence of *Wolbachia* and *Spiroplasma* in the three *D. melanogater* strains used in our experiments (Fig. 1). We used gene-specific primers to amplify nucleotide sequences of the gene coding for the *Wolbachia* surface protein (*Wsp*) (160 bp) [43], and the *Spiroplasma* gene *DnaA* (138 bp) [44]. We also performed PCR control reactions to amplify a 360 bp fragment of the *D. melanogaster* housekeeping gene *RpL32* [45]. We confirmed that one *D. melanogaster* strain contained both *Wolbachia* and *Spiroplasma* endosymbionts (W + S+), one strain contained *Wolbachia* bacteria only (W + S-), and one strain contained no endosymbiotic bacteria (W-S-).

### *Wolbachia* and *Spiroplasma* do not affect the survival response of *D. melanogaster* to *P. luminescens* infection

**Fig. 2:**
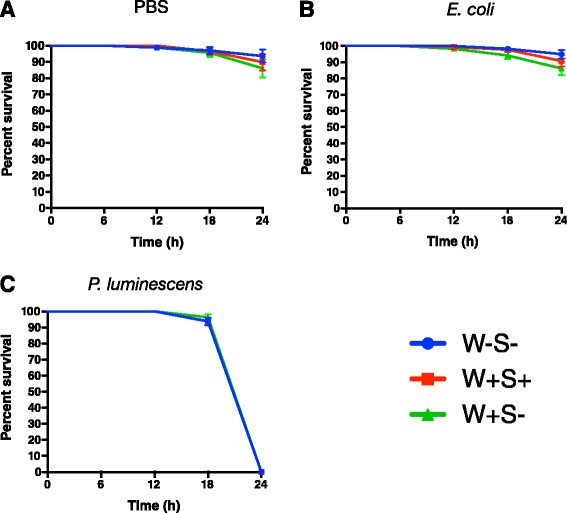
Survival of *D. melanogaster* flies carrying or lacking endosymbionts following bacterial infection. Survival of 7-10 day old flies lacking *Wolbachia* and *Spiroplasma* (W-S-), flies carrying both endosymbionts (W + S+), and flies carrying *Wolbachia* only (W + S-) following intrathoracic injection with (**a**) sterile PBS (septic injury control), (**b**) *E. coli* bacteria (strain K12), or (**c**) *P. luminescens* bacteria (strain TT01). Survival was monitored for 24 h at 6-h intervals. Data analysis was performed using Log-Rank test (GraphPad Prism5 software) and the values are the percent survival of the infected flies. The means from three independent experiments are shown and bars represent standard errors

We initially investigated the survival response of *D. melanogaster* flies carrying or lacking endosymbionts to infection by *P. luminescens* bacteria. We also performed injections with non-pathogenic *E. coli* and PBS buffer (Fig. 2). For flies injected with either *E. coli* or PBS, survival at the 24-h time-point was very high and not affected by the endosymbiont status of the flies (W + S+, W + S-, or W-S-; log-rank test, *P >* 0.05 in both cases; Fig. 2a and b). Infection with the insect pathogenic bacterium *P. luminescens* resulted in death of all strains within 24 h, but again, there was no significant effect of the endosymbionts on fly survival (log-rank test, *P >* 0.05; Fig. 2c). These results show that the presence of *Wolbachia* alone or together with *Spiroplasma* in *D. melanogaster* does not affect survival of the flies in response to infection with either non-pathogenic *E. coli* bacteria nor the entomopathogen *P. luminescens*.

### Presence of endosymbionts can alter bacterial load in *D. melanogaster* following infection with non-pathogenic bacteria

**Fig. 3:**
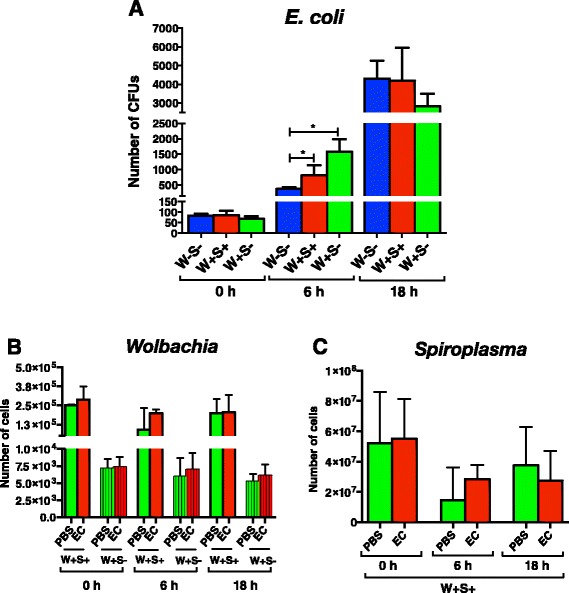
*E. coli* load and endosymbiont titers in infected *D. melanogaster* flies carrying or lacking endosymbionts*. E. coli* bacteria (strain K12) or 1x sterile PBS were injected into *D. melanogaster* flies lacking *Wolbachia* and *Spiroplasma* (W-S-), flies carrying both endosymbionts (W + S+), and flies carrying *Wolbachia* only (W + S-). **a** Colony Forming Units (CFUs) for *E. coli*, (**b**) number of cells for *Wolbachia*, and (**c**) number of cells for *Spiroplasma* at 0, 6 and 18 h after infection were determined by quantitative PCR. Data analysis was performed by unpaired two-tailed *t*-test and significant differences are indicated by an asterisk (*P < 0.05). Bars show the means from three independent experiments and error bars represent standard deviations

To examine changes in bacterial load following infection with a non-pathogenic strain of *E. coli*, we injected *E. coli* K12 cells into W + S+, W + S-, and W-S- adult flies and estimated number of CFU at 6 and 18 h post infection. We found that at 6 h there were signifIcantly higher numbers of *E. coli* cells in W + S+ and W + S- flies than in W-S- flies (*P <* 0.05, Fig. 3a), although there were no significant differences in *E. coli* numbers among the different strains at 0 and 18 h time-points (*P >* 0.05, Fig. 3a).

We then tested whether infection with non-pathogenic *E. coli* bacteria affects the number of *Wolbachia* and *Spiroplasma* endosymbionts in *D. melanogaster* flies. Using the same samples and a PCR approach, we found no significant differences in *Wolbachia* numbers between the *E. coli* infected strains and the PBS injected controls for any of the time-points used in the experiments (*P >* 0.05, Fig. 3b). We also estimated *Spiroplasma* numbers in W + S+ flies injected with PBS buffer or non-pathogenic *E. coli* bacteria, and we found no significant changes at any time-point (*P >* 0.05, Fig. 3c).

### Presence of endosymbionts does not alter bacterial load in *D. melanogaster* following *P. luminescens* infection

**Fig. 4:**
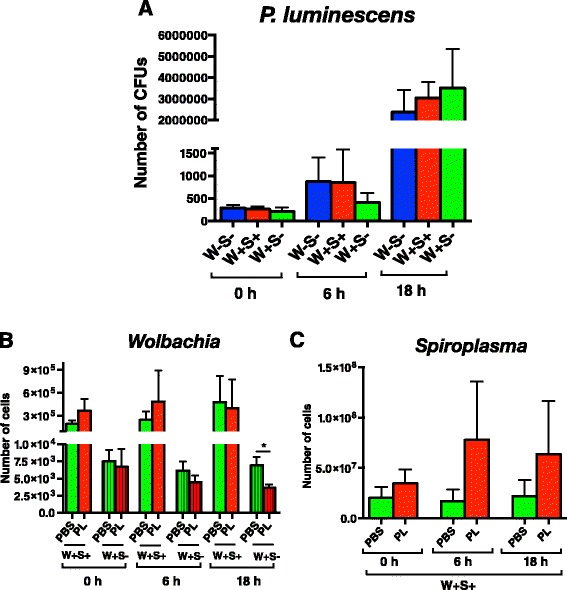
*P. luminescens* load and endosymbiont titers in infected *D. melanogaster* flies carrying or lacking endosymbionts*. P. luminescens* pathogenic bacteria (strain TT01) or 1x sterile PBS were injected into *D. melanogaster* adult flies lacking *Wolbachia* and *Spiroplasma* (W-S-), flies carrying both endosymbionts (W + S+), and flies carrying *Wolbachia* only (W + S-). **a** Colony Forming Units (CFUs) for *P. luminescens*, (**b**) number of cells for *Wolbachia*, and (**c**) number of cells for *Spiroplasma* at 0, 6 and 18 h after infection were determined by quantitative PCR. Data analysis was performed by unpaired two-tailed *t*-test and significant differences are indicated by an asterisk (*P < 0.05). Bars show the means from three independent experiments and error bars represent standard deviations

To test whether the presence of *Wolbachia* and *Spiroplasma* in *D. melanogaster* affects replication of insect pathogenic bacteria, we injected adult flies with *P. luminescens* and monitored bacterial load at two time-points post infection. Although we found that *P. luminescens* CFU increased steadily at 6 and 18 h post infection, there were no significant differences in *P. luminescens* numbers among the W + S+, W + S-, and W-S- fly strains (*P >* 0.05, Fig. 4a). We also evaluated numbers of *Wolbachia* endosymbionts in *P. luminescens* infected flies and PBS injected flies and found that at 18 h post injection, W + S- flies contained significantly higher numbers of *Wolbachia* than the uninfected controls (*P <* 0.05, Fig. 4b). However, there were no significant changes in *Spiroplasma* numbers in the W + S+ strain upon injection with PBS or the pathogenic bacteria (*P >* 0.05, Fig. 4c).

### Changes in immune gene transcription in *D. melanogaster* flies carrying *Wolbachia* and *Spiroplasma* upon bacterial infection

**Fig. 5:**
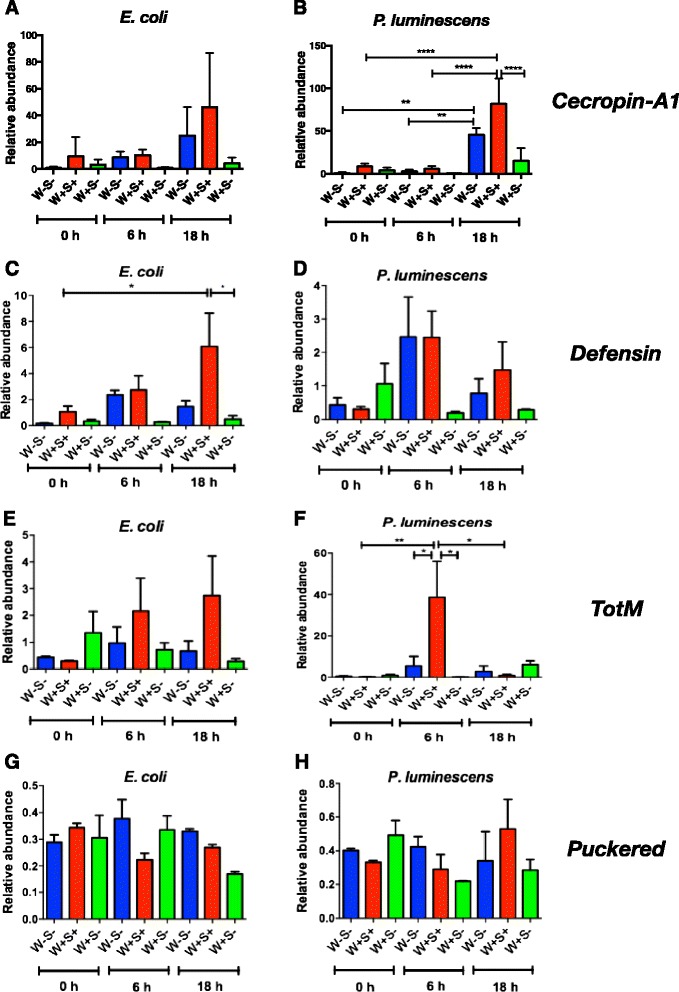
Transcription of immune signaling pathway genes in flies carrying or lacking endosymbionts following bacterial infection. Gene transcription levels for *Cecropin-A1* (**a**, **b**), *Defensin* (**c**, **d**), *Turandot* (*Tot*) *M* (**e**, **f**) and *Puckered* (**g**, **h**) in *D. melanogaster* adult flies without *Wolbachia* and *Spiroplasma* (W-S-), with both endosymbionts (W + S+), and with *Wolbachia* only (W + S-) at 0, 6 and 18 h after infection with *E. coli* (strain K12) or *P. luminescens* (strain TT01). Gene transcription levels are shown as relative abundance of transcripts normalized to gene *RpL32* and expressed as a ratio compared to flies injected with sterile PBS (negative control). Values represent the means from three biological replicates and error bars represent standard deviations. ****P < 0.0001; **P < 0.01; *P < 0.05 (one way analysis of variance with a Tukey *post hoc* test, GraphPad Prism5 software)

An important aspect of the innate immune system of *D. melanogaster* is the transcriptional induction of AMP genes in response to bacterial infections. The Imd pathway is activated by diaminopimelic acid (DAP)-type peptidoglycan that is found in all Gram-negative and certain Gram-positive bacteria, while the Toll pathway is activated by Lys-type peptidoglycan found in most other Gram-positive bacteria [46]. Here we used qRT-PCR to determine the induction of immune signaling pathways in *Drosophila* strains carrying or lacking endosymbionts at the time of injection with *E. coli* or *P. luminescens* and at two time-points post bacterial infection (Fig. 5). For this, we used gene-specific primers to estimate the transcription of *Cecropin-A1* as a readout for the Imd pathway, *Defensin* for the Toll pathway, *Turandot M* (*TotM*) for the Janus kinase and signal transducer and activator of transcription (Jak/Stat) pathway and *Puckered* for the c-Jun N-terminal kinase (JNK) pathway [47–50]. The non-pathogenic bacteria *E. coli* altered gene transcription in all three *D. melanogaster* strains compared to uninfected controls (Fig. 5a, c, e, g). We also found that transcription of *Defensin* in flies containing *Wolbachia* and *Spiroplasma* was significantly higher at 18 h post-infection compared to the 0 h time-point (*P <* 0.05, Fig. 5c). However, *Defensin* transcription was significantly higher in flies containing both endosymbionts compared to those containing *Wolbachia* only at 18 h post infection (*P <* 0.05, Fig. 5c). For gene transcription in flies infected with the pathogen *P. luminescens*, we found that *Cecropin-A1* transcription significantly increased at 18 h post infection in flies lacking endosymbionts (*P <* 0.01, Fig. 5b) and in those containing both *Wolbachia* and *Spiroplasma* (*P <* 0.0001, Fig. 5b). Also, at 18 h post infection with *P. luminescens*, flies carrying both endosymbionts showed significantly stronger *Cecropin-A1* transcription compared to those containing *Wolbachia* only (*P <* 0.0001, Fig. 5b). *TotM* transcription in flies carrying both endosymbionts was significantly higher at 6 h post infection with *P. luminescens* compared to the other two time-points (*P <* 0.05, Fig. 5f). In addition, *TotM* transcription was significantly higher in flies containing both *Wolbachia* and *Spiroplasma* compared to flies containing *Wolbachia* only or no endosymbionts at 6 h post-infection (*P <* 0.05, Fig. 5f). There were no significant differences in *Defensin* and *Puckered* gene transcription between the three fly strains following infection with *P. luminescens* bacteria at any of the time-points tested in this study (*P >* 0.05, Fig. 5b, d, h). These results suggest that the presence of both *Wolbachia* and *Spiroplasma* endosymbionts in *D. melanogaster* flies can alter the transcription of certain immune-related genes following infection with non-pathogenic bacteria or the insect-specific pathogen *P. luminescens*.

### *Wolbachia* and *Spiroplasma* do not alter phagocytosis in *D. melanogaster*

**Fig. 6:**
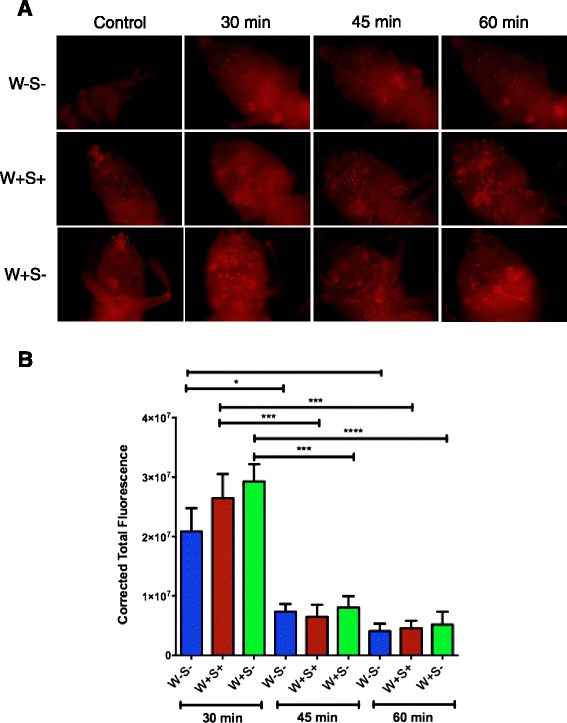
Phagocytosis in *D. melanogaster* flies carrying or lacking endosymbiotic bacteria. **a** Representative images of phagocytosis in *D. melanogaster* adult flies without *Wolbachia* and *Spiroplasma* (W-S-), with both endosymbionts (W + S+), and with *Wolbachia* only (W + S-) at 30, 45 and 60 min after injection of lipophilized pHrodo-labeled *E. coli* particles. Control treatments involved injections with DEPC-treated water. Images were taken using fluorescence microscopy and 10x magnification. **b** Corrected total fluorescence in the three *D. melanogaster* flies at 30, 45 and 60 min following injection of pHrodo-labeled *E. coli*. Images were processed in ImageJ and corrected total fluorescence was estimated by measuring relative amounts of fluorescence, which included estimations of the resulting area, mean fluorescence of background and integrated density. The experiment was repeated three times with 6-12 flies for each treatment. ****P < 0.0001; ***P < 0.001; **P < 0.01; *P < 0.05 (one way analysis of variance with a Tukey *post hoc* test, GraphPad Prism5 software)

We estimated phagocytic ingestion in *D. melanogaster* strains carrying or lacking endosymbionts by injecting inactivated unopsonized fluorogenic *E. coli* particles into adult flies and using fluorescence microscopy (Fig. 6a). We found that phagocytosis of *E. coli* particles in *D. melanogaster* flies was not significantly affected by the presence of *Wolbachia* endosymbionts alone or in combination with *Spiroplasma* endosymbionts at any time-point after injection of the fluorescent particles (*P >* 0.5, Fig. 6b). However, we noticed that regardless of whether flies contained *Wolbachia* only or both *Wolbachia* and *Spiroplasma*, phagocytosis levels were significantly higher (P values ranged from < 0.1 to < 0.0001) at 30 min after injection of the *E. coli* particles compared to the 45 and 60 min time-points. These results support the notion that *Wolbachia* and *Spiroplasma* endosymbionts do not affect the phagocytic function of *D. melanogaster* adult flies.

## Discussion

Recent studies have examined the involvement of endosymbiotic bacteria in the immune response of different *Drosophila* species against infections by various bacterial pathogens [35–37]; however, here we investigated for the first time changes in immune signalling and function in *D. melanogaster* flies against the virulent insect-specific pathogen *P. luminescens*. These bacteria are known to be extremely pathogenic to a wide range of insect species because they can secrete a vast array of toxins and they can employ mechanisms to disarm the insect immune system [38, 41]. Current survival results are in agreement with recent findings reporting that *D. melanogaster* flies carrying or lacking *Wolbachia* endosymbionts were able to survive similarly upon infection with the Gram-negative bacteria *Pseudomonas aeruginosa*, *Serratia marcescens*, and *Erwinia carotovora* [35]; however, *D. melanogaster* flies carrying a naturally occurring strain of *Spiroplasma* and without the presence of other ensosymbionts proved more sensitive to the Gram-negative pathogens *E. carotovora* and *Enterobacter cloacae*, but not to the Gram-positive pathogen *Enterococcus faecalis* [37].

Although *Wolbachia* alone or in combination with *Spiroplasma* endosymbionts failed to provide protection to *D. melanogaster* against *P. luminescens* infection, we investigated whether the pathogens replicated at similar or different rates in the three fly strains and whether differential replication of these bacteria would affect endosymbiont titers in the infected flies. We were unable to find changes in *P. luminescens* load among the different strains, which shows that *Wolbachia* does not influence the growth of this pathogen in the fly. Previous studies have also failed to find significant differences in pathogen load between fly strains carrying or lacking *Wolbachia* endosymbionts [35–37]. Our data also suggest that the presence of *Spiroplasma* in *D. melanogaster* in addition to *Wolbachia* endosymbionts does not affect the replication of *P. luminescens* and therefore the presence of those endosymbiotic microbes in the fly is not essential for altering the growth of this pathogen. Strikingly, there was a drop in *Wolbachia* titers in flies carrying those endosymbionts alone after infection with *P. luminescens*, which could imply that these endosymbiotic bacteria can be directly or indirectly targeted by the pathogen.

Reduced numbers of *E. coli* cells in flies with no endosymbionts compared to flies containing both endosymbionts or *Wolbachia* only at early times after infection indicates that the absence of endosymbiotic bacteria may control more efficiently the early stages of an infection by non-pathogenic bacteria. Alternatively, the presence of endosymbionts in the fly could provide a favorable environment that could promote the early replication of exogenous non-pathogenic bacteria, which do not affect insect survival and they are ultimately cleared by the fly immne system [51]. The increase in *E. coli* numbers in flies containing endosymbiotic bacteria at 6 h post infection is not accompanied by changes in the numbers of *Wolbachia* cells. These results imply that although the existence of *Wolbachia* in *D. melanogaster* may confer a positive effect on the replication of exogenous non-pathogenic bacteria, this effect is not reciprocal as it fails to stimulate the propagation of those endosymbionts in the fly. Previously, clean injury of wild-type flies or infection with the Gram-positive *Micrococcus luteus* or the Gram-negative *E. carotovora* stimulated *Spiroplasma* growth [37]; however, in our experiments we did not observe any changes in *Spiroplasma* cell numbers in flies injected with the bacteria or PBS. Therefore, we conclude that distinct types of bacterial infections can differentially influence the titers of certain endosymbionts in *D. melanogaster* flies.

Our current results show that *Wolbachia* endosymbionts in combination with *Spiroplasma* significantly induce the activation of Imd signaling, but not Toll and JNK signaling, in *D. melanogaster* upon injection with *P. luminescens* bacteria. Activation of the Imd pathway in flies carrying *Wolbachia* and *Spiroplasma* upon infection with *P. luminescens*, but not in flies containing *Wolbachia* only or no endosymbionts suggests that the presence of both endosymbiotic bacteria is essential to trigger Imd signaling in response to the pathogen. Interestingly, previous studies have shown lack of Imd pathway activation in flies containing *Spiroplasma* only upon infection with the Gram-negative bacteria *E. carotovora* [37], or *Wolbachia* only upon infection with three Gram-negative bacterial pathogens [35]. We speculate that Imd upregulation in flies carrying both *Wolbachia* and *Spiroplasma* in response to *P. luminescens* infection is probably a distinct effect to this pathogen and possibly to other insect-specific pathogenic bacteria. The molecular basis of this effect is currently unclear and will be a subject of our future research. In addition, we have previously observed low levels of *Defensin* mRNA in *P. luminescens* infected flies [52], which is in agreement with the inability of Gram-negative bacteria containing DAP-type peptidoglycan to induce Toll signaling in *D. melanogaster* [46]. Here we were curious to know whether the presence of endosymbionts could alter the transcriptional levels of *Defensin* that is partially regulated by the Toll pathway. Our current findings confirm our previous results that *Defensin* is transcribed at low levels upon infection with *P. luminescens* [52], and additionally show that the presence of *Wolbachia* alone or together with *Spiroplasma* bacteria does not affect Toll signaling in response to this pathogen. Up-regulation of *Defensin* by *E. coli* in flies containing both *Wolbachia* and *Spiroplasma* but not in those containing either *Wolbachia* only or no endosymbiotic bacteria is an unexpected yet intriguing result, because it suggests that *Spiroplasma* endosymbionts that lack a cell wall and the molecular patterns that trigger immune pathway activation are somehow able to induce Toll signaling upon challenge with non-pathogenic bacteria. The molecular processes that lead to Toll activation in *Spiroplasma*-containing flies are currently unknown and beg for further investigation. In contrast to our findings, previous studies have failed to show activation of a systemic immune response by *Spiroplasma* endosymbionts in infected or uninfected *D. melanogaster* adult flies [37, 53, 54]. We further suspect that *P. luminescens* infection in combination with the occurrence of both endosymbionts in these flies is likely to increase stress conditions that could lead to early overactivation of Jak/Stat signaling, although induction of this pathway in W + S+ flies does not alter pathogen load and the survival response of the flies to the pathogen.

We then examined the potential effect of endosymbionts on the *D. melanogaster* cellular immune response, which mainly involves the activity of circulating macrophage-like insect blood cells called hemocytes that engulf microbes through phagocytosis [55]. We did not perform infections with stained *P. luminescens* cells because this pathogen has been shown previously to interfere with the cellular immune response by disrupting phagocytosis and other hemocyte-related immune functions such as hemocyte aggregation, nodulation and encapsulation [41]. We found no evidence that *Wolbachia* endosymbionts alone or together with *Spiroplasma* affect phagocytosis of *E. coli* particles in *D. melanogaster* flies. Because *Wolbachia* are intracellular bacteria that are found in various tissues but mainly occupy the reproductive organs of their hosts [13, 19], it would be rather unlikely that these endosymbionts can interact with insect hemocytes to alter the phagocytic ability of their host. However, *Spiroplasma* endosymbionts are primarily found in the hemolymph [12], and although they could come into contact with phagocytic cells, their potential interaction does not seem to affect phagocytosis of inactive bacterial particles. It has also been shown earlier that genetic ablation of phagocytes in *D. melanogaster* adult flies does not reduce *Spiroplasma* titers in the hemolymph [37].

Future work will focus on the role of endosymbiotic bacteria in the immune response of *D. melanogaster* flies against infection by the mutualistic partner of *P. luminescens* bacteria, the parasitic nematode *H. bacteriophora* [39]. Interestingly, recent studies have shown that the mushroom-feeding flies *D. neotestacea* harboring *Spiroplasma* endosymbionts have increased tolerance to their natural nematode parasite *Howardula aoronymphium* [56], and that the number of worms decreases in flies carrying *Spiroplasma,* but not in those lacking the endosymbionts [57]. In addition, it will be of particular interest to test the participation of endosymbionts against entomopathogenic nematodes and their associated bacteria in *D. melanogaster* larvae, which have a distinct immune response compared to the adult fly [58, 59].

## Conclusions

In this study we demonstrate that injection of the virulent entomopathogenic bacterium *P. luminescens* into *D. melanogaster* adult flies carrying *Wolbachia* endosymbionts alone or *Wolbachia* and *Spiroplasma* bacteria together can affect the transcriptional activation of certain immune-related genes and *Wolbachia* titers, but not pathogen load. However, infection of *D. melanogaster* flies carrying both endosymbionts with a non-pathogenic strain of *E. coli* can affect the transcriptional activation of AMP genes and bacterial burden, but not endosymbiont numbers. We further show that the presence of endosymbiotic bacteria in the fly does not alter phagocytosis of inactive bioparticles. Finally we find that changes in AMP gene transcription and bacterial load do not change the survival of *D. melanogaster* strains containing *Wolbachia* only or both endosymbiotic bacteria compared to those lacking the endosymbionts following infection with *P. luminescens* or *E. coli*.

## Abbreviations

AMP: Antimicrobial peptide
ANOVA: Analysis of variance
cDNA: Complementary DNA
CFU: Colony forming units
DEPC: Diethylpyrocarbonate
IJ: Infective juvenile
Imd: Immune deficiency
Jak/Stat: Janus kinase and signal transducer and activator of transcription
JNK: c-Jun N-terminal kinase
LB: luria-bertani
MAPK: Mitogen-activated protein kinase
NF-κB: Nuclear factor-kappa-B
PBS: Phosphate buffered saline
PCR: Polymerase chain reaction
qRT-PCR: Quantitative real-time PCR
TAE: Tris-acetate-EDTA
Tot: Turandot

## References

[1] Uvell H, Engström Y (2007). A multilayered defense against infection: combinatorial control of insect immune genes. Trends Genet.

[2] Buchon N, Silverman N, Cherry S (2014). Immunity in *Drosophila melanogaster*--from microbial recognition to whole-organism physiology. Nat Rev Immunol.

[3] Ganesan S, Aggarwal K, Paquette N, Silverman N (2011). NF-κB/Rel proteins and the humoral immune responses of *Drosophila melanogaster*. Curr Top Microbiol Immunol.

[4] Marmaras VJ, Lampropoulou M (2009). Regulators and signalling in insect haemocyte immunity. Cell Signal.

[5] Eleftherianos I, Revenis C (2011). Role and importance of phenoloxidase in insect hemostasis. J Innate Immun.

[6] Kim SH, Lee WJ (2014). Role of DUOX in gut inflammation: lessons from *Drosophila* model of gut-microbiota interactions. Front Cell Infect Microbiol.

[7] Davies SA, Dow JA (2009). Modulation of epithelial innate immunity by autocrine production of nitric oxide. Gen Comp Endocrinol.

[8] Dionne MS, Schneider DS (2008). Models of infectious diseases in the fruit fly *Drosophila melanogaster*. Dis Model Mech.

[9] Valanne S (2014). Functional genomic analysis of the *Drosophila* immune response. Dev Comp Immunol.

[10] Su Q, Zhou X, Zhang Y (2013). Symbiont-mediated functions in insect hosts. Commun Integr Biol.

[11] Hilgenboecker K, Hammerstein P, Schlattmann P, Telschow A, Werren JH (2008). How many species are infected with *Wolbachia*?--A statistical analysis of current data. FEMS Microbiol Lett.

[12] Haselkorn TS (2010). The *Spiroplasma* heritable bacterial endosymbiont of *Drosophila*. Fly.

[13] Werren JH, Baldo L, Clark ME (2008). *Wolbachia*: master manipulators of invertebrate biology. Nat Rev Microbiol.

[14] Saridaki A, Bourtzis K (2010). *Wolbachia*: more than just a bug in insects genitals. Curr Opin Microbiol.

[15] Regassa LB, Gasparich GE (2006). *Spiroplasmas*: evolutionary relationships and biodiversity. Front Biosci.

[16] Gross J, Bhattacharya D (2009). Mitochondrial and plastid evolution in eukaryotes: an outsiders’ perspective. Nat Rev Genet.

[17] Douglas AE (2011). Lessons from studying insect symbioses. Cell Host Microbe.

[18] Weiss BL, Maltz M, Aksoy S (2012). Obligate symbionts activate immune system development in the tsetse fly. J Immunol.

[19] Eleftherianos I, Atri J, Accetta J, Castillo JC (2013). Endosymbiotic bacteria in insects: guardians of the immune system?. Front Physiol.

[20] Brownlie JC, Johnson KN (2009). Symbiont-mediated protection in insect hosts. Trends Microbiol.

[21] McGraw EA, O’Neill SL (2004). *Wolbachia pipientis*: intracellular infection and pathogenesis in *Drosophila*. Curr Opin Microbiol.

[22] Bourtzis K, Pettigrew MM, O’Neill SL (2000). *Wolbachia* neither induces nor suppresses transcripts encoding antimicrobial peptides. Insect Mol Biol.

[23] Teixeira L, Ferreira A, Ashburner M (2008). The bacterial symbiont *Wolbachia* induces resistance to RNA viral infections in *Drosophila melanogaster*. PLoS Biol.

[24] Hedges LM, Brownlie JC, O’Neill SL, Johnson KN (2008). *Wolbachia* and virus protection in insects. Science.

[25] Osborne SE, Leong YS, O’Neill SL, Johnson KN (2009). Variation in antiviral protection mediated by different *Wolbachia* strains in *Drosophila simulans*. PLoS Pathog.

[26] Shaw AE, Veronesi E, Maurin G, Ftaich N, Guiqen F, Rixon F, Ratinier M, Mertens P, Carpenter S, Palmarini M, Terzian C, Arnaud F. *Drosophila melanogaster* as a model organism for bluetongue virus replication and tropism. J Virol. 2012;86:9015–24. 10.1128/JVI.00131-12 PMC3416142 22674991

[27] Hedges LM, Yamada R, O’Neill SL, Johnson KM (2012). The small interfering RNA pathway is not essential for *Wolbachia*-mediated antiviral protection in *Drosophila melanogaster*. Appl Environ Microbiol.

[28] Chrostek E, Marialva MS, Esteves SS, Weinert LA, Martinez J, Jiggins FM, Teixeira L. *Wolbachia* variants induce differential protection to viruses in *Drosophila melanogaster*: a phenotypic and phylogenomic analysis. PLoS Genet. 2013;9:e1003896. 10.1371/journal.pgen.1003896 PMC3861217 24348259

[29] Chrostek E, Marialva MS, Yamada R, O’Neill SL, Teixeira L (2014). High anti-viral protection without immune upregulation after interspecies *Wolbachia* transfer. PLoS One.

[30] Martinez J, Longdon B, Bauer S, Chan YS, Miller WJ, Bourtzis K, Teixeira L, Jiggins FM. Symbionts commonly provide broad spectrum resistance to viruses in insects: a comparative analysis of *Wolbachia* strains. PLoS Pathog. 2014;10:e1004369. 10.1371/journal.ppat.1004369 PMC4169468 25233341

[31] Ferreira ÁG, Naylor H, Esteves SS, Pais IS, Martins NE, Teixeira L (2014). The Toll-dorsal pathway is required for resistance to viral oral infection in *Drosophila*. PLoS Pathog.

[32] Fytrou A, Schofield PG, Kraaijeveld AR, Hubbard SF (2006). *Wolbachia* infection suppresses both host defence and parasitoid counter-defence. Proc Biol Sci.

[33] Xie J, Vilchez I, Mateos M (2010). *Spiroplasma* bacteria enhance survival of *Drosophila hydei* attacked by the parasitic wasp *Leptopilina heterotoma*. PLoS One.

[34] Xie J, Tiner B, Vilchez I, Mateos M (2011). Effect of the *Drosophila* endosymbiont *Spiroplasma* on parasitoid wasp development and on the reproductive fitness of wasp-attacked fly survivors. Evol Ecol.

[35] Wong ZS, Hedges LM, Brownlie JC, Johnson KN (2011). *Wolbachia*-mediated antibacterial protection and immune gene regulation in *Drosophila*. PLoS One.

[36] Rottschaefer SM, Lazzaro BP (2012). No effect of *Wolbachia* on resistance to intracellular infection by pathogenic bacteria in *Drosophila melanogaster*. PLoS One.

[37] Herren JK, Lemaitre B (2011). *Spiroplasma* and host immunity: activation of humoral immune responses increases endosymbiont load and susceptibility to certain Gram-negative bacterial pathogens in *Drosophila melanogaster*. Cell Microbiol.

[38] Waterfield NR, Ciche T, Clarke D (2009). *Photorhabdus* and a host of hosts. Ann Rev Microbiol.

[39] Ciche T. The biology and genome of Heterorhabditis bacteriophora. WormBook. 2007. doi:10.1895/wormbook.1.135.1. 10.1895/wormbook.1.135.1 PMC4781484 18050499

[40] Ffrench-Constant RH, Dowling A, Waterfield NR (2007). Insecticidal toxins from *Photorhabdus* bacteria and their potential use in agriculture. Toxicon.

[41] Eleftherianos I, Ffrench-Constant RH, Clarke DJ, Dowling AJ, Reynolds SE (2010). Dissecting the immune response to the entomopathogen *Photorhabdus*. Trends Microbiol.

[42] Ventura IM, Martins AB, Lyra ML, Andrade CA, Carvalho KA, Klaczko LB (2012). *Spiroplasma* in *Drosophila melanogaster* populations: prevalence, male-killing, molecular identification, and no association with *Wolbachia*. Microb Ecol.

[43] Newton IL, Sheehan KB (2015). Passage of *Wolbachia pipientis* through mutant *Drosophila melanogaster* induces phenotypic and genomic changes. Appl Environ Microbiol.

[44] Kageyama D, Anbutsu H, Watada M, Hosokawa T, Shimada M, Fukatsu T (2006). Prevalence of a non-male-killing spiroplasma in natural populations of *Drosophila hydei*. Appl Environ Microbiol.

[45] Rugjee KN, Roy Chaudhury S, Al-Jubran K, Ramanathan P, Matina T, Wen J, Brogna S. Fluorescent protein tagging confirms the presence of ribosomal proteins at *Drosophila* polytene chromosomes. PeerJ. 2013;1:e15. 10.7717/peerj.15 PMC3629075 23638349

[46] Tanji T, Hu X, Weber AN, Ip YT (2007). Toll and IMD pathways synergistically activate an innate immune response in *Drosophila melanogaster*. Mol Cell Biol.

[47] Kaneko T, Silverman N (2014). Bacterial recognition and signalling by the *Drosophila* IMD pathway. Cell Microbiol.

[48] Imler JL, Bulet P (2005). Antimicrobial peptides in *Drosophila*: structures, activities and gene regulation. Chem Immunol Allergy.

[49] Brun S, Vidal S, Spellman P, Takahashi K, Tricoire H, Lemaitre B (2006). The MAPKKK Mekk1 regulates the expression of *Turandot* stress genes in response to septic injury in *Drosophila*. Genes Cells.

[50] McEwen DG, Peifer M (2005). Puckered, a *Drosophila* MAPK phosphatase, ensures cell viability by antagonizing JNK-induced apoptosis. Development.

[51] Lemaitre B, Hoffmann J (2007). The host defense of *Drosophila melanogaster*. Annu Rev Immunol.

[52] Castillo JC, Shokal U, Eleftherianos I, Castillo JC, Shokal U, Eleftherianos I (2012). Immune gene transcription in *Drosophila* adult flies infected by entomopathogenic nematodes and their mutualistic bacteria. J Insect Physiol.

[53] Hurst GD, Anbutsu H, Kutsukake M, Fukatsu T (2003). Hidden from the host: *Spiroplasma* bacteria infecting *Drosophila* do not cause an immune response, but are suppressed by ectopic immune activation. Insect Mol Biol.

[54] Anbutsu H, Fukatsu T (2010). Evasion, suppression and tolerance of *Drosophila* innate immunity by a male-killing *Spiroplasma* endosymbiont. Insect Mol Biol.

[55] Vlisidou I, Wood W (2015). *Drosophila* blood cells and their role in immune responses. FEBS J.

[56] Jaenike J, Unckless R, Cockburn SN, Boelio LM, Perlman SJ (2010). Adaptation via symbiosis: recent spread of a *Drosophila* defensive symbiont. Science.

[57] Jaenike J, Brekke TD (2011). Defensive endosymbionts: a cryptic trophic level in community ecology. Ecol Lett.

[58] Fellous S, Lazzaro BP (2010). Larval food quality affects adult (but not larval) immune gene expression independent of effects on general condition. Mol Ecol.

[59] Fellous S, Lazzaro BP (2010). Potential for evolutionary coupling and decoupling of larval and adult immune gene expression. Mol Ecol.

